# Prevalence of Robust Older Adults Among Homebound Older Adults

**DOI:** 10.1093/geroni/igaf122.1028

**Published:** 2025-12-31

**Authors:** Bruce Leff, Katherine Ornstein, Mary Louise Pomeroy, Christine Ritchie, Jennifer Reckrey, Orla Sheehan

**Affiliations:** Johns Hopkins University School of Medicine, Baltimore, Maryland, United States; Johns Hopkins University, Baltimore, Maryland, United States; Johns Hopkins Bloomberg School of Public Health, Baltimore, Maryland, United States; Massachusetts General Hospital, Boston, Massachusetts, United States; NYU Grossman School of Medicine, New York, New York, United States; Royal College of Surgeons in Ireland, Dublin, Dublin, Ireland

## Abstract

Over seven million U.S. older adults are homebound. The majority of homebound older adults are unable to access office-based primary care. Most descriptions of the homebound imply that they are universally frail, but this hypothesis hasn’t been empirically tested. We aimed to determine in a nationally representative sample of U.S. older adults the prevalence and degrees of frail and robust among the homebound using the physical frailty phenotype (PFP), and compare sociodemographic, clinical, and social characteristics of non-frail homebound to prefrail and frail homebound and using self-reported health care utilization data compare utilization across frailty subgroups. Using representative data from the National Health and Aging Trends study (NHATS) study (2011-2019, 2021-2023) we assessed the weighted prevalence of frailty among persons continuously homebound over 2 years and found that 46.3% were frail, 47.9% were prefrail and 8.1% were robust. Compared to those who were frail or prefrail, robust homebound older adults had fewer comorbidities, were more likely to be female, had higher education, income and less mental health disorders, functional and cognitive impairment. Prior work using claims-based frailty algorithms reported higher prevalence rates for the robust homebound raising a concern for misclassification in prior work due to an inability for this population to access care and generate claims. In our cohort the prevalence of the prefrail is similar to the prevalence of frailty creating the potential for targeted care interventions appropriate to the needs of the pre-frail homebound to prevent progression to frailty in this vulnerable population.

